# Excessive fuel availability amplifies the FTO-mediated obesity risk: results from the TUEF and Whitehall II studies

**DOI:** 10.1038/s41598-017-15744-4

**Published:** 2017-11-14

**Authors:** Róbert Wagner, Ádám G. Tabák, Ellen Fehlert, Louise Fritsche, Benjamin A. Jaghutriz, Róbert J. Bánhegyi, Sebastian M. Schmid, Harald Staiger, Fausto Machicao, Andreas Peter, Hans-Ulrich Häring, Andreas Fritsche, Martin Heni

**Affiliations:** 1 Department of Internal Medicine IV, Division of Endocrinology, Diabetology, Nephrology, Vascular Disease and Clinical Chemistry, University Hospital of Tübingen, Tübingen, Germany; 2 Institute for Diabetes Research and Metabolic Diseases of the Helmholtz Centre Munich at the University of Tübingen (IDM), Tübingen, Germany; 3 German Center for Diabetes Research (DZD), Neuherberg, Germany; 4 Department of Epidemiology and Public Health, University College London, London, UK; 5 1st Department of Medicine, Semmelweis University Faculty of Medicine, Budapest, Hungary; 6 Pándy Kálmán Hospital, Gyula, Hungary; 7 Department of Internal Medicine I, Division of Endocrinology, Diabetology, and Metabolism, University Hospital of Lübeck, Lübeck, Germany; 8 Interfaculty Centre for Pharmacogenomics and Pharma Research at the Eberhard Karls University Tübingen, Tübingen, Germany; 9 Institute of Pharmaceutical Sciences, Department of Pharmacy and Biochemistry, Eberhard Karls University Tübingen, Tübingen, Germany; 10 Institute of Experimental Genetics, Helmholtz Centre Munich, German Research Center for Environmental Health, Neuherberg, Germany

## Abstract

Variation in *FTO* is the most important common genetic determinant of body weight. Altered energy metabolism could underlie this association. We hypothesized that higher circulating glucose or triglycerides can amplify the *FTO* impact on BMI. In 2671 subjects of the TUEF study, we investigated the interaction effect of fasting glucose and triglyceride levels with rs9939609 in *FTO* on BMI. We analysed the same interaction effect by longitudinally utilizing mixed effect models in the prospective Whitehall II study. In TUEF, we detected an interaction effect between fasting glucose and fasting triglycerides with rs9939609 on BMI (p = 0.0005 and p = 5 × 10^−7^, respectively). The effect size of one risk allele was 1.4 ± 0.3 vs. 2.2 ± 0.44 kg/m² in persons with fasting glucose levels below and above the median, respectively. Fasting triglycerides above the median increased the per-allele effect from 1.4 ± 0.3 to 1.7 ± 0.4 kg/m^2^. In the Whitehall II study, body weight increased by 2.96 ± 6.5 kg during a follow-up of 13.5 ± 4.6 yrs. Baseline fasting glucose and rs9939609 interacted on weight change (p = 0.009). Higher fasting glucose levels may amplify obesity-risk in *FTO* carriers and lead to an exaggerated weight gain over time. Since weight gain perpetuates metabolic alterations, this interplay may trigger a vicious circle that leads to obesity and diabetes.

## Introduction

Due to its increasing global prevalence, obesity has become a major health problem worldwide. Since it is strongly linked to insulin resistance, obesity increases the disease burden of type 2 diabetes. The pathogenesis of obesity comprises an intricate interplay of genetic and environmental factors. For the genetic contribution, around a hundred single nucleotide polymorphisms (SNPs) are reported to be associated with BMI^1^. However, the aggregate influence of these known variants explains the heritability of obesity to a small extent (~2.7%) only^1^. Genetic effects can show striking variation, depending on the metabolic environment which, in turn, reflects lifestyle or other genetic factors. Interactions between environmental factors and genetic loci might therefore account for some of the variability^2^. The impact of the common genetic variant in *FTO*, the polymorphism most strongly and consistently associated with obesity, is modulated by such factors. Physical activity, aerobic fitness, and diet interact with the *FTO* variant on obesity or the change of body weight over time^3–6^. For other diabetes-related genetic variants, such as *TCF7L2*, glycemia is an important interacting factor modulating the SNP’s effect^7^. We have already demonstrated that the risk allele of *FTO* is associated with higher food intake^8^, altered processing of food signals in the brain^9^ and with insulin resistance of the human brain^10^. Recent data suggest that the variation in *FTO* also has an impact on the transcription factors that regulate adipocyte development^11^. Indeed, adipocytes from *FTO* risk allele carriers have an altered energy homeostasis: they store energy more efficiently and produce less excess heat^11^. We thus hypothesized that higher plasma levels of energy substrates, namely glucose and triglycerides, could intensify this increased lipid storage, thereby causing excessive weight gain, particularly in subjects carrying the risk allele in *FTO*.

## Methods

All methods were performed in accordance with the relevant guidelines and regulations.

### Subjects

#### TUEFF

The Tübingen Family Study for type 2 diabetes (TUEF) is designed to establish a thoroughly phenotyped cohort of a population at increased risk of type 2 diabetes. To this end, subjects with known prediabetes (without known diabetes), a family history of diabetes, or obesity are continuously recruited to evaluate metabolic status with respect to glucose and lipid metabolism. The Ethics Committee of the Medical Faculty of the University of Tübingen approved the study protocol. Written informed consent was obtained from all participating individuals. Further details on the study have already been published^12^.

#### Whitehall-II

Data from the occupational Whitehall II cohort were accessed by a data sharing agreement. Details of the study have already been published^13^. The study was approved by the Joint UCL/UCLH Committees on the Ethics of Human Research (Committee Alpha). All participants provided written informed consent. The Whitehall II study was initiated in 1985 and recruited 10,308 participants (3,413 women) aged 35–55 years with a response rate of 73% from 20 London based Civil Service departments. The initial visit (phase 1) included a clinical examination and a self-administered questionnaire in 1985–88. During follow-up, 5-yearly clinical examinations were performed (Phase 3: 1991–94, Phase 5: 1997–99, Phase 7: 2002–04, and Phase 9: 2007–09) and additional postal questionnaire only phases were conducted (Phase 2:1988–90, Phase 4: 1995–96, Phase 6: 2001, and Phase 8: 2006). As 75 g oral glucose tolerance tests (OGTT) were first performed in phase 3, this provides the baseline for the current analysis. Disclosed data was available for 5067 white participants for phases 3, 5, 7 and 9, amounting to a total of 24,029 person-examinations. After removing missing or non-fasted glucose measurements^14^, missing weight or genotyping information and participants with known diabetes, we gained a full dataset comprising 16,307 person-examinations.

#### Oral glucose tolerance test (OGTT) and laboratory analyses

In the TUEF study, all participants received a 75-g glucose solution (Accu-Check Dextro, Roche) at 8 a.m. following an overnight fast. Venous blood was obtained through an indwelling venous catheter before and 30, 60, 90 and 120 minutes after glucose ingestion. The procedure of OGTT in the Whitehall II study was already described in detail^14^.

Glucose was analysed in both the TUEF and the Whitehall II study using an YSI glucose analyser (Yellow Springs Instruments).

Fasting triglyceride concentrations were measured by a standard colorimetric method on a Bayer analyser (Bayer HealthCare) in TUEF, and by a Cobas Fara centrifugal analyser (Roche Diagnostics System) in Whitehall II^15^.

#### Genotyping

Following appropriate cell lysis, protein precipitation and washing in the TUEF study, the SNP rs9939609 was genotyped from blood samples using the MassARRAY platform (Sequenom). Genotyping of Whitehall II participants was described earlier^16^.

#### Statistical analysis

Data are presented as median and interquartile range or mean ± SD. To determine whether glycemia or triglycerides modulate the effect of the SNP on body weight and BMI, linear regression modelling was performed with the least squares method, including the interaction term SNP × environmental factor. Age and sex were included as covariates in all linear regression models. From the linear regression models, the effect sizes are given as effect estimates (estimate). Records with missing data were excluded. The *FTO* SNP rs9939609 genotype was modelled according to the additive inheritance model; the SNP variable indicating the number of risk alleles. Longitudinal data in the Whitehall II cohort was investigated by testing fold-change BMI between adjacent visits. Linear mixed models were fitted, adjusting for the random effects subject and time since the beginning of the study. Fixed effect variables comprised sex, BMI on the previous visit, age, age-squared, elapsed time between the observations, fasting glucose on the previous visit, and the genotype, with all interactions of the latter three variables. The analyses were performed with R 3.3.2 (R Core Team), using the lme4 package for mixed models.

### Data availability statement

The dataset of the TUEF study is not publicly available due to ethical reasons concerning the participants’ informed consents, but is available from the corresponding author on appropriate request. The availability of the Whitehall-II data is subject to an individual data-sharing agreement.

## Results

### TUEF Study

**Figure 1:**
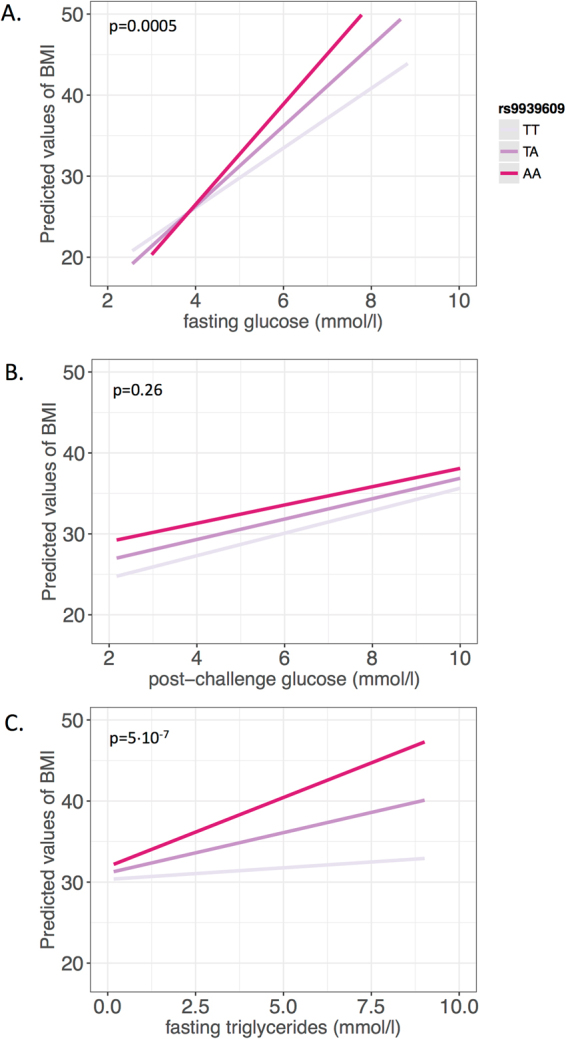
Linear regression models plotting the predicted values of BMI against fasting glucose (**A**), post-challenge glucose (**B**), and fasting plasma triglycerides (**C**) in interaction with the *FTO* SNP rs9939609 in the TUEF study. Colors indicate genotypes of rs9939609. The p-values are given for the respective interaction terms in models adjusted for sex, age, and either fasting triglycerides in panel A or fasting glucose in panel C.

Participants of the cross-sectional TUEF study (N = 2671) had a median BMI above 28 kg/m^2^, and the interquartile ranges indicated a high proportion of subjects with higher BMI (see Table 1). As anticipated, genetic variation in *FTO* was strongly associated with BMI (p = 1.7·10^−10^), even after adjusting for sex and age (p = 2.9·10^−10^). The effect size on BMI amounted to 1.7 ± 0.3 kg/m^2^ per risk allele. We fitted linear regression models to assess the impact of both glucose levels and plasma triglycerides on the association of the genetic variant in *FTO* with BMI. We tested the interaction terms between *FTO* × fasting glucose, *FTO* × post-challenge glucose and *FTO* × fasting plasma triglycerides on BMI. In all models, age and sex were included as additional covariates. Genetic variation in *FTO* revealed significant interaction with fasting glucose in models with (p = 0.0005) and without (p = 0.003) additional adjustment for fasting plasma triglycerides. This translated to a 1.78 ± 0.73 kg/m^2^ higher BMI associated with each 1 mmol/l higher glucose value in the presence of the AA risk genotype than in the TT genotype. This interaction remained significant even after additional adjustment for insulin sensitivity (p = 0.003). In similar models, fasting plasma triglycerides showed interaction effects with *FTO* on BMI with (p = 5·10^−7^) and without (p = 0.0002) additional adjustment for fasting glucose. The presence of the AA risk genotype rather than the TT genotype implicated a 2.11 ± 0.51 kg/m^2^ higher BMI associated with each 1 mmol/l higher triglyceride value. However, no interaction of *FTO* with post-challenge glucose was observed (p = 0.3) (see Fig. 1).

**Table 1:** Baseline data from the TUEF and Whitehall II* studies.

study	TUEF (N = 2671)		Whitehall II* (N = 4966)	
weight (kg)	85	(70.7–103.4)	75.1	(67.2–83.2)
BMI (kg/m²)	28.5	(24.2–35.7)	24.8	(22.8–27.0)
**sex distribution**	**females 64%**		**females 26%**	
age (years)	41	(30–52)	49.5	(45.0–55.7)
fasting glucose (mmol/l)	5.2	(4.8–5.6)	5.2	(4.9–5.5)
fasting triglycerides (mmol/l)	1.15	(0.8–1.7)	1.16	(0.8–1.7)
*FTO* rs9939609 genotypes (AA/AT/TT in % of all)	32/50/18		36/48/16	

Given are medians and interquartile ranges or percentage.

*Whitehall II data are presented for participants in the first available phase.

**Figure 2:**
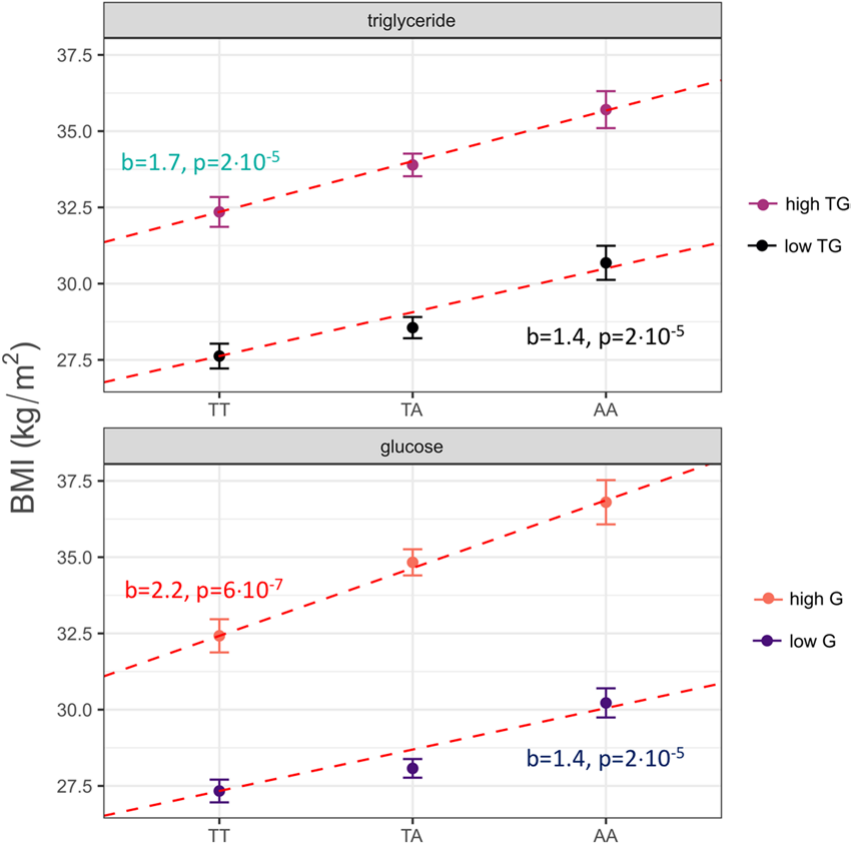
Relationship of *FTO* rs9939609 genotypes and BMI in subgroups stratified at the medians of fasting triglycerides (low TG, high TG) and fasting glucose (low G, high G). BMI per genotype is shown in each stratum as least squares mean with standard error (adjusted for sex, age, and either fasting triglycerides (in the glucose strata) or fasting glucose (in the triglyceride strata). The linear regression estimate, slope (b) with corresponding p-value (p), is shown by dashed lines.

We next stratified the study population at the median of fasting glucose levels into *low-glucose* (<5.17 mmol/l) and *high-glucose* (≥5.17 mmol/l) groups. Genetic variation in *FTO* exhibited a lower effect size in the *low-glucose* group (estimate = 1.4 ± 0.3 kg/m^2^, p = 6·10^−6^) than in the *high-glucose* group (estimate = 2.2 ± 0.44 kg/m^2^, p = 6·10^−7^). A similar stratification was performed using *low-triglyceride* (<1.15 mmol/l) and *high-triglyceride* (≥1.15 mmol/l) groups. The impact of *FTO* on BMI was also lower in the *low-triglyceride* group (estimate = 1.4 ± 0.3 kg/m^2^, p = 2·10^−5^) than in the *high-triglyceride* group (estimate = 1.7 ± 0.4 kg/m^2^, p = 2·10^−5^). All models were adjusted for sex, age, plasma triglycerides, and fasting glucose, respectively. The data are also shown in Fig. 2.

### Whitehall II Study

**Figure 3:**
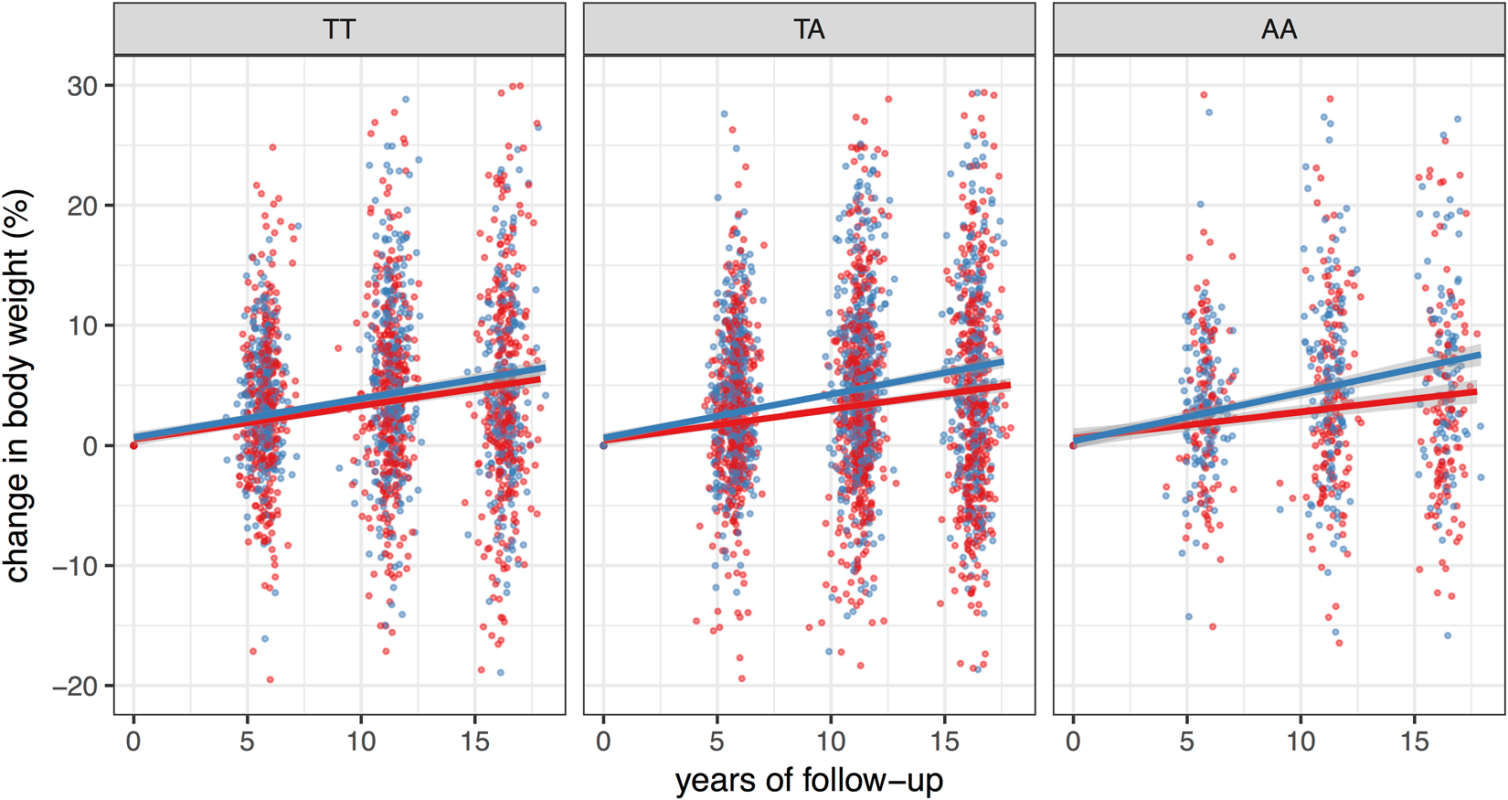
Interaction effect of glycemia and rs9939609 in *FTO* on change of body weight in the longitudinal Whitehall-II study (p = 0.04). Panels show participants carrying different *FTO* rs9939609 genotypes, colours indicate quartiles of average fasting glucose per subject over the full observation period (red: bottom quartile, fasting glucose 4.07–4.9 mmol/l, blue: top quartile, fasting glucose 5.44–16 mmol/l). Lines indicate linear fits per glucose quartile with 95% confidence intervals in the respective colours.

In this longitudinal cohort, we tested whether fasting glucose and triglyceride concentrations modulate the impact of the *FTO* SNP on BMI over time. During the observation period of 13.5 ± 4.6 years, the 4966 participants involved displayed a mean weight gain of 2.96 ± 6.5 kg, translating to a BMI change of 1.42 ± 2.2 kg/m^2^, i.e. a 5.7 ± 8.6% relative increase in BMI. BMI and diabetes incidence at the last observation, stratified by *FTO* genotypes, is provided in Supplementary Table 1. Using a linear mixed model, we modelled the BMI change between consecutive visits as a function of the *FTO* SNP and the fasting glucose levels assessed during the previous visit. The models also comprised the covariates sex, age, age-squared, and elapsed time since the last observation. Fasting glucose during the previous observation significantly interacted with *FTO* on BMI change (*b* = 0.002 ± 0.0008, *p* = 0.04, Supplementary Figure 1). This translates to a 0.2 percentage-points higher BMI increase per risk-allele for 1 mmol/l higher fasting glucose. We also ascertained an inversely directed significant interaction between elapsed time since the last observation, fasting glucose in the last observation, and *FTO* (*b* = −0.0019 ± 0.0008, *p* = 0.02), suggesting that the interaction effect diminishes over time. No comparable interaction was evident for fasting plasma triglycerides (p = 0.6). Moreover, post-challenge glucose levels did not modulate the impact of genetic *FTO* variation on weight gain (p = 0.67). To allow for robust visualization of the data, we computed individual average fasting glucose levels over all available time points and stratified this variable into quartiles. A comparison of the lowest and the highest mean fasting glucose quartiles showed that mean fasting glucose modulated the impact of the *FTO* genotype over the total observation period (p = 0.04). This is indicative of a progressively higher weight gain in subjects who have higher average fasting glucose levels and who are carriers of the *FTO* risk allele (see Fig. 3).

## Discussion

In this study, we investigated whether plasma glucose or triglyceride levels modulate the effect of the BMI-associated genetic variant in *FTO*. In two independent study populations, we demonstrated that fasting glucose and *FTO* interact to increase BMI and weight change over time. This interaction effect was also observed in the cross-sectional TUEF population with a broad range of BMI. However, post-challenge glucose does not interact with *FTO* genotype to increase obesity.

Gene × environment interactions are proposed to contribute to substantial parts of the hitherto genetically unexplained heritability of metabolic traits such as obesity or diabetes^17^. A recent family-based study showed the presence of significant gene × environment interactions for BMI involving environmental factors such as sex, age, alcohol intake and other dietary habits^18^. While glycemia is also an outcome of genetic and environmental factors, it is nevertheless an important attribute of the metabolic environment which, in turn, modulates multiple cellular processes. We have already shown that glucose levels interact with what is to date the most important diabetes-associated common SNP in *TCF7L2* to affect insulin secretion^19^.

Numerous biological mechanisms may underlie the interaction between glucose and *FTO* detected in this study. Recent research indicates that *FTO* variation has a profound effect on adipocyte energy handling via the transcription factors IRX3 and IRX5^11,20^. Increased expression of these transcription factors leads to cell-autonomous changes during adipocyte development, causing a phenotype that is associated with increased lipid storage and reduced energy loss via thermogenesis^11^. Both circulating glucose and triglycerides are important substrate resources for lipid storage in adipocytes. Adipocytes synthesize triglycerides from glucose through de novo lipogenesis^21^, while circulating triglycerides are hydrolysed by lipoprotein-lipase and then re-esterified to triglycerides within the adipocytes^22^.

Abundance of energy carriers might thus augment the impact of *FTO* genotype by increasing substrate supply. One might therefore expect basal energy expenditure to be lower in *FTO* risk allele carriers. Despite several studies on this topic, however, there is still no convincing evidence that *FTO* has any impact on energy expenditure in humans^23^. However, we and others have shown that *FTO* risk allele carriers have an increased caloric intake^8^. In line with this, there is growing evidence that variation in *FTO*, the gene with the highest expression in the central nervous system, has an impact on central nervous appetite regulation^9,24,25^ and brain insulin sensitivity^10^. These processes in the human brain may involve the central dopaminergic system since genetically determined dopamine receptor density modulates *FTO* effects even further^26,27^. We speculate that insulin resistance and appetite dysregulation associated with the risk allele of *FTO* could also contribute to the observed shift in the glucose-BMI regression line, as shown in Fig. 1A, thus explaining this interaction. Elevated glucose levels are known to suppress appetite, induce satiety and reduce food intake^28,29^. Since the association of *FTO* variation with brain activity is more pronounced in the context of elevated glucose^9^, higher glucose levels may enhance the impact of *FTO* on body weight also via the brain.

Limitations of this work include the different study populations, different measurement methods and differences in OGTT conditions which could potentially reduce the comparability of the two studies. A longitudinal analysis was available in Whitehall II only. Furthermore, circulating glucose and triglycerides does not necessarily indicate cellular energy availability to a full extent.

By demonstrating that glycemia and possibly also triglyceride levels interact with *FTO* to modulate body weight, our work provides additional evidence for the importance of gene × environment interactions in the pathogenesis of obesity. Although the underlying biological mechanism of this interaction cannot be elucidated by our data, our findings fit well into the known biological context. Our data suggest that individuals with increased fasting glucose levels have an even higher risk for weight gain if the strongest common genetic variant in the *FTO* gene is present. Since weight gain is known to further accelerate metabolic deterioration, this interplay may result in a vicious circle and thus lead to a more pronounced obese state and promote diabetes risk. Effective pharmacological and non-pharmacological glucose-lowering strategies may therefore be required to boost weight loss, particularly in *FTO* risk-allele carriers suffering from prediabetes or diabetes.

## Electronic supplementary material

Supplementary Table 1 and Figure 1
