# Supplementary material for: Excessive fuel availability amplifies the FTO-mediated obesity risk: results from the TUEF and Whitehall II studies
Source: Sci Rep. 2017 Nov 14;7:15486. doi: 10.1038/s41598-017-15744-4 (PMC5686126; doi:10.1038/s41598-017-15744-4)
Supplement: Supplementary file 1 — Supplementary Table 1 and Figure 1 [file 41598_2017_15744_MOESM1_ESM.doc]

# Supplementary Information

Excessive fuel availability amplifies the FTO-mediated obesity risk.

Results from the TUEF and Whitehall II studies

Róbert Wagner^1,2,3^, Ádám G. Tabák^4,5^, Ellen Fehlert^1,2,3^, Louise Fritsche^1,2,3^, Benjamin A. Jaghutriz^1,2,3^, Róbert J. Bánhegyi^6^, Sebastian M. Schmid^3,7^, Harald Staiger^2,3,8, 9,10^, Fausto Machicao^10^, Andreas Peter^1,2,3^, Hans-Ulrich Häring^1,2,3^, Andreas Fritsche^1,2,3^, Martin Heni^1,2,3^

## Supplementary Table 1

Cross-sectional data on the Whitehall-II cohort at the last observation stratified on genotypes (median and interquartile range).

|  | **Genotypes of rs9939609 in *FTO*** | | |
| --- | --- | --- | --- |
|  | TT | TA | AA |
| **BMI (kg/m^2^)** | 25.8 (23.6-28.5) | 26.2 (23.7-28.9) | 26.6 (24.1-29.1) |
| **Incident diabetes (%)** | 5.4 | 6.1 | 5.2 |

## Supplementary Figure 1

Marginal effects plot from the mixed model showing the interaction of fasting glucose and the *FTO* variant rs9939609 (additive model, the numbers indicate copies of the obesity risk allele A) on percent change of BMI between two visits in the Whitehall-II study. Colored lines are regression lines for three different glucose levels from the mixed model (red: mean, blue: mean-SD, yellow: mean+SD).
